# An Exploration of Caregiver Health and Wellbeing

**DOI:** 10.1002/alz.084426

**Published:** 2025-01-09

**Authors:** Maria E Anderson

**Affiliations:** ^1^ University of Saint Joseph, West Hartford, CT USA

## Abstract

**Background:**

Many individuals with health problems and/or disabilities are largely dependent on the help of an informal caregiver, most often a family member with whom they live (CDC Report, 2018). A recent report by the Alzheimer’s Association (2023) found that, compared with caregivers of people without dementia, twice as many caregivers of those with dementia have reported significant emotional, financial, and physical difficulties.

Despite the important role that caregivers have in our society, research on potential factors that may buffer the negative impacts of caregiving has been lacking. A small number of studies conducted in this area have shown, for example, that holding positive beliefs toward caregiving may contribute meaningfully to the care individuals with disabilities and health problems receive (e.g., Kim & Bolkan, 2018). But generally, studies on this topic tend to focus on healthcare professionals (e.g., Zimmerman et al., 2005), not informal caregivers (Brodaty & Donkin, 2009)

Previous work on older adults has shown that holding positive beliefs can meaningfully impact one’s health, quality of life, and even lifespan (e.g., Levy et al., 2004; Levy et al., 2018). This has also been demonstrated experimentally; activating age‐related stereotypes in healthy older adults has been shown to improve memory performance in domains generally thought to decline with age (Levy et al., 1996).

**Methods:**

The current study seeks to add to our understanding of the impact that a caregiver’s own positive or negative beliefs toward their role can have on their own well‐being. Our study used a scrambled‐word task to expose caregivers to either positive or negative stereotypes related to caregiving. We then measured the perception of burden, confidence in their role as a caregiver, and perception of their quality of life.

**Results:**

Preliminary results showed that a prime of negative language (e.g., “struggle” or “guilt”) may be powerful enough to cause individuals to perceive their burden as greater compared to those primed with positive language (“wisdom” and “useful”).

**Conclusion:**

This follow‐up study will explore more deeply the effects that perceptions around support and confidence have on an individual’s feeling of positivity toward caregiving.

